# Tumor volume increases the predictive accuracy of prognosis for gastric cancer: A retrospective cohort study of 3409 patients

**DOI:** 10.18632/oncotarget.14859

**Published:** 2017-01-27

**Authors:** Zhen Liu, Peng Gao, Shushang Liu, Gaozan Zheng, Jianjun Yang, Li Sun, Liu Hong, Daiming Fan, Hongwei Zhang, Fan Feng

**Affiliations:** ^1^ Division of Digestive Surgery, Xijing Hospital of Digestive Diseases, The Fourth Military Medical University, 710032, Xi’an, Shaanxi Province, China; ^2^ Department of Radiation Medicine, Faculty of Preventive Medicine, The Fourth Military Medical University, 710032, Xi’an, Shaanxi Province, China

**Keywords:** gastric cancer, tumor volume, prognosis, predictive accuracy

## Abstract

Tumor diameter or T stage does not reflect the actual tumor burden and is not able to estimate accurate prognosis of gastric cancer. The current study aimed to evaluate the prognostic value of tumor volume (V) for gastric cancer. A total of 3409 enrolled gastric cancer patients were randomly divided into training set (*n* = 1705) and validation set (*n* = 1704). Tumor volume was calculated by the formula V = Tumor diameter × (T stage)^2^/2. The survival predictive accuracy and prognostic discriminatory ability between different variables and staging systems were analyzed. Four optimal cutoff points for V were obtained in training set (3.5, 8.6, 25.0, 45.0, all *P* < 0.001). V stage was significantly associated with tumor location, macroscopic type, differentiation degree, tumor diameter, T stage, N stage, vessel invasion, neural invasion and TNM stage (all *P* < 0.001). V stage was an independent prognostic factor both in training and validation set. V stage showed better predictive accuracy and prognostic discriminatory ability than tumor diameter and T stage. VNM staging system also have advantages in predictive accuracy and prognostic discriminatory ability than TNM staging system. The VNM multivariable model represent good agreement between the predicted survival and actual survival. In conclusion, tumor volume was significantly associated with clinicopathological features and prognosis of gastric cancer. In comparison with TNM staging system, VNM staging system could improve the predictive accuracy and prognostic discriminatory ability for gastric cancer.

## INTRODUCTION

Although the incidence of gastric cancer has significantly decreased worldwide, it is still the second most common malignancy in China [1]. Thus, identification of its risk factors for prognosis remains greatly important to clinicians. A variety of factors have been adequately analyzed in order to evaluate their predictive value of prognosis for gastric cancer, including tumor diameter [2], T stage [3], N stage [4], tumor markers [5, 6] and other novel indexes [7–9].

Till now, the most commonly used classification is TNM staging system including T stage, N stage and distant metastasis, which was recommended by American Joint Committee on Cancer (AJCC) [10] and Japanese Gastric Cancer Association (JGCA) [11]. However, the tumor diameter, as an important prognostic factor which was demonstrated in many other tumors [12–15] as well as gastric cancer [16], has not been included in the TNM staging system yet. Thus, in present study, we defined a new index—tumor volume (V) by the formula V = Tumor diameter × (T stage)^2^/2, and investigated the prognostic value of tumor volume and VNM for gastric cancer.

## RESULTS

### General features of gastric cancer patients

There were 2662 males (78.1%) and 747 females (21.9%). The patient age ranged from 20 to 90 years (median, 58; mean, 57). The follow up time ranged from 1 to 75 months (median, 24.9; mean, 28.1). The 1-, 3- and 5-year overall survival rate was 89.0%, 66.6% and 57.9%, respectively. There were 1705 patients in training set and 1704 patients in validation set. The clinicopathological characteristics were comparable between training and validation set (Table 1).

**Table 1:** Clinicopathological characteristics of patients in training and validation set

Characteristics	Training set						Validation set						*P* value
	V1	V2	V3	V4	V5	*P* value	V1	V2	V3	V4	V5	*P* value	
Age						0.311						0.461	0.989
≤ 60	213	110	324	226	139		203	107	321	240	140		
> 60	127	68	214	174	110		124	72	206	183	108		
Gender						0.576						0.068	0.051
Male	268	149	425	312	201		242	152	403	325	185		
Female	72	29	113	88	48		85	27	124	98	63		
Tumor location						< 0.001						< 0.001	0.850
Upper third	53	48	195	153	90		61	48	181	159	85		
Middle third	58	22	84	58	50		52	27	98	64	41		
Lower third	218	96	230	151	67		202	99	222	156	89		
**Upper-middle or middle-lower**	11	12	29	38	42		12	5	26	44	33		
Macroscopic type						< 0.001						< 0.001	0.387
Early stage	309	2	0	0	0		291	1	0	0	0		
Bormann I	6	22	50	34	29		7	15	39	36	19		
Bormann II	11	119	164	76	44		19	124	173	65	35		
Bormann III	1	24	265	212	131		2	25	251	255	137		
Bormann IV	2	4	40	56	32		0	9	38	41	50		
**Differentiation degree**						< 0.001						< 0.001	0.736
**Well differentiated**	101	19	44	20	8		114	14	44	20	4		
**Moderately differentiated**	90	44	160	86	48		88	68	146	100	37		
**Poorly differentiated**	136	105	304	264	164		114	92	321	271	185		
**Mucinous or signet ring cell**	10	9	26	25	28		11	5	16	30	22		
Tumor diameter*						< 0.001						< 0.001	0.954
≤ 2.5 cm	232	47	52	0	0		230	52	58	0	0		
2.5–4.3 cm	96	129	243	79	2		75	126	252	87	2		
4.3–5.5 cm	7	0	206	103	4		15	0	186	107	3		
> 5.5 cm	5	2	37	218	243		7	1	31	229	243		
T stage						< 0.001						< 0.001	0.699
T1	326	2	0	0	0		306	1	0	0	0		
T2	14	169	79	0	0		20	174	78	0	0		
T3	0	6	389	218	14		1	4	395	229	8		
T4a	0	1	69	180	220		0	0	53	193	230		
T4b	0	0	1	2	15		0	0	1	1	10		
N stage						< 0.001						< 0.001	0.587
N0	288	86	158	67	22		274	88	155	69	26		
N1	32	40	138	64	24		32	41	146	88	26		
N2	14	25	114	91	61		16	30	105	96	43		
N3a	5	24	102	125	82		5	16	92	121	103		
N3b	1	3	26	53	60		0	4	29	49	50		
Vessel invasion						< 0.001						< 0.001	0.874
Positive	45	65	209	233	187		51	60	214	235	180		
Negative	182	58	166	80	40		175	55	139	102	49		
Neural invasion						< 0.001						< 0.001	0.347
Positive	62	87	314	278	218		70	75	302	313	215		
Negative	128	38	63	37	8		119	39	56	24	16		
TNM stage						< 0.001						< 0.001	0.239
IA	279	1	0	0	0		260	1	0	0	0		
IB	39	81	29	0	0		41	85	35	0	0		
IIA	17	45	140	41	3		20	43	132	42	0		
IIB	5	24	140	72	20		4	29	143	71	27		
IIIA	0	26	105	65	24		2	21	102	110	27		
IIIB	0	1	96	139	64		0	0	98	120	43		
IIIC	0	0	28	83	138		0	0	17	80	151		
VNM stage						< 0.001						< 0.001	0.963
IA	288	0	0	0	0		274	0	0	0	0		
IB	32	86	0	0	0		32	88	0	0	0		
IIA	14	40	161	0	0		16	41	166	0	0		
IIB	6	25	143	67	0		5	30	139	69	0		
IIIA	0	27	107	64	0		0	20	106	88	0		
IIIB	0	0	127	91	46		0	0	116	96	52		
IIIC	0	0	0	178	203		0	0	0	170	196		

Tumor diameter*: Tumor diameter was divided into 4 subgroups according to the 3 optimal cutoff points calculated by X-tile software (Supplementary Figure 1).

### Definition of V stage and VNM stage

**Figure 1:**
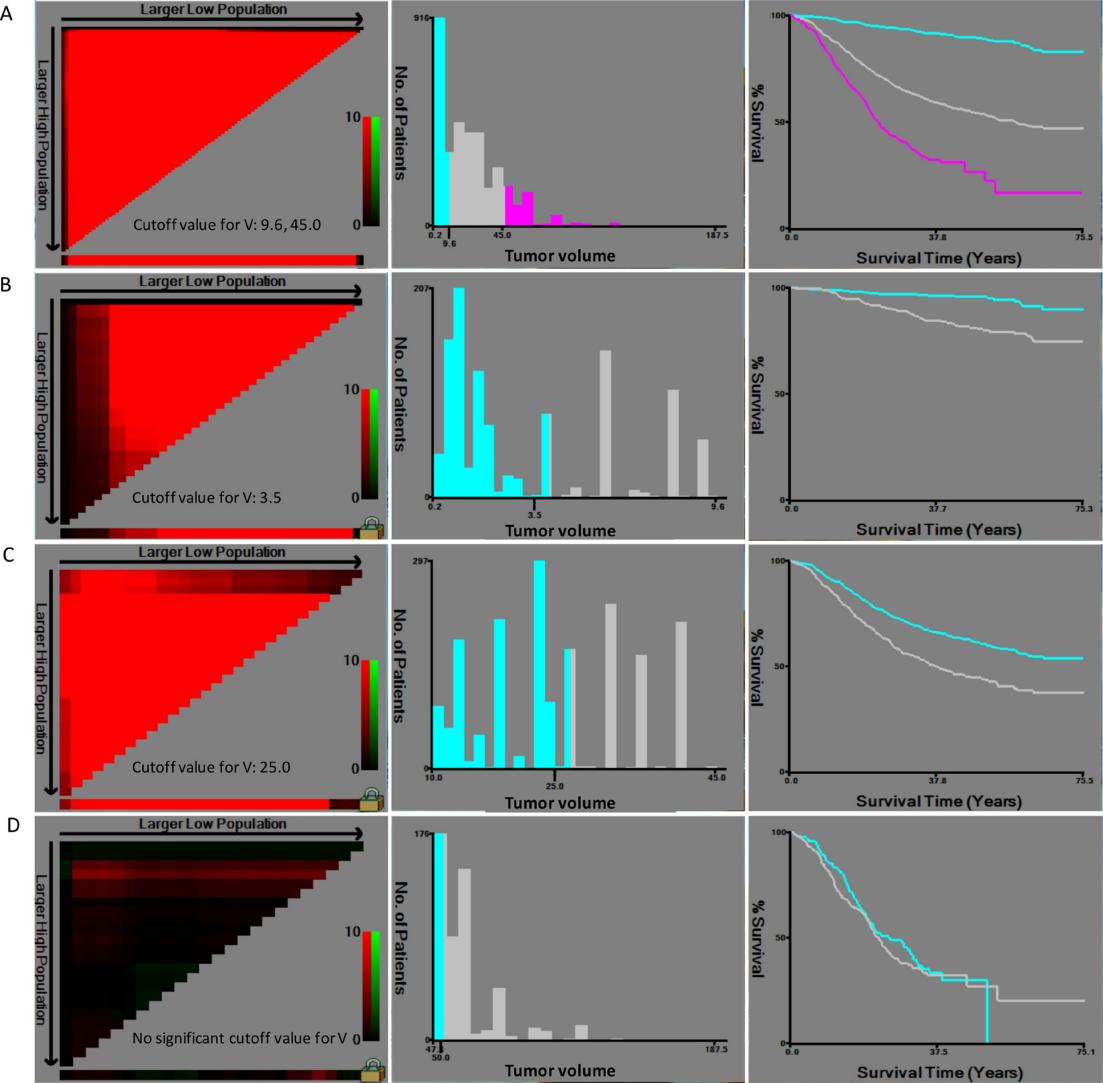
Calculation of cutoff points of tumor volume by X-tile in training set (**A**) Three subgroups were built according to the 2 optimal cutoff points (9.6, 45.0, *P <* 0.001); (**B**) Two subgroups were built according to the optimal cutoff point (3.5, *P <* 0.001) for patients with tumor volume between 0 and 9.6. (**C**) Two subgroups were built according to the optimal cutoff point (25.0, *P <* 0.001) for patients with tumor volume between 9.6 and 45.0. (**D**) No cutoff point was obtained for patients with tumor volume exceed 45.0.

Tumor volume was calculated by the formula V = Tumor diameter × (T stage)^2^/2 (1 represents T1 stage, 2 represents T2 stage, 3 represents T3 stage, 4 represents T4a stage, and 5 represents T4b stage). The 4 optimal cutoff points of tumor volume (all *P <* 0.05) in training set were showed in Figure 1. Then, V stage was defined according to the 4 cutoff points: V1 (≤ 3.5), V2 (3.5–8.6), V3 (8.6–25.0), V4 (25.0–45.0) and V5 (> 45.0). VNM system was designed as combination of V stage, N stage and M stage on the basis of 7th edition of AJCC cancer staging manual.

The correlation between V stage and other factors were analyzed in Table 1. Both in training and validation set, V stage was found to be significantly associated with tumor location (*P <* 0.001), macroscopic type (*P <* 0.001), differentiation degree (*P <* 0.001), tumor diameter (*P <* 0.001), T stage (*P <* 0.001), N stage (*P <* 0.001), vessel invasion (*P <* 0.001), neural invasion (*P <* 0.001) and TNM stage (*P <* 0.001). Compared with the small tumor volume-patients, patients with larger tumor volume were found more frequently in Borrmann type III or IV, having a higher proportion in poor differentiation, in advanced T stage and N stage, in positive vessel and neural invasion and in advanced TNM stage.

### Prognostic value of V stage in gastric cancer

Prognostic predictors were identified by univariate and multivariate analysis in training set (Table 2). Age (*P* = 0.025), tumor location (*P* = 0.004), macroscopic type (*P <* 0.001), differentiation degree (*P <* 0.001), tumor diameter (*P <* 0.001), T stage (*P <* 0.001), N stage (*P <* 0.001), V stage (*P <* 0.001), vessel invasion (*P <* 0.001) and neural invasion (*P <* 0.001) were risk factors for prognosis of gastric cancer. Multivariate analysis (Table 2) showed that age (*P* = 0.016), macroscopic type (*P* = 0.001), N stage (*P <* 0.001) and V stage (*P <* 0.001) were independent prognostic factors for gastric cancer.

**Table 2:** Univariate and multivariate analysis of overall survival in training set

Characteristics	Univariate analysis			Multivariate analysis			C-index	AIC
	β	HR (95% CI)	*P* value	β	HR (95% CI)	*P* value		
Age	0.203	1.225 (1.026–1.464)	0.025	0.283	1.327 (1.053–1.671)	0.016	0.528	3936.8
Gender	0.017	1.017 (0.818–1.265)	0.879				0.499	3935.5
Tumor location	0.003	1.003 (1.001–1.006)	0.004				0.516	3937.0
Macroscopic type	0.540	1.716 (1.566–1.879)	< 0.001	0.257	1.292 (1.109–1.507)	0.001	0.653	3832.8
**Differentiation degree**	0.422	1.525 (1.352–1.720)	< 0.001				0.593	3894.7
Tumor diameter	0.632	1.882 (1.721–2.058)	< 0.001				0.686	3835.3
T stage	0.736	2.087 (1.889–2.306)	<0.001				0.681	3780.3
N stage	0.657	1.930 (1.798–2.072)	< 0.001	0.561	1.753 (1.576–1.949)	< 0.001	0.736	3698.2
V stage	0.681	1.975 (1.820–2.144)	< 0.001	0.340	1.405 (1.235–1.599)	< 0.001	0.715	3768.2
Vessel invasion	1.087	2.966 (2.282–3.855)	< 0.001				0.614	3871.8
Neural invasion	1.237	3.445 (2.395–4.955)	< 0.001				0.579	3880.2

HR: Hazard ratio; CI: Confidence interval.

The prognostic value of V stage was also analyzed in validation set using the cutoff points from training set (Table 3). V stage was still the independent prognostic factor for gastric cancer in validation set (*P* = 0.045).

**Table 3:** Univariate and multivariate analysis of overall survival in validation set

Characteristics	Univariate analysis			Multivariate analysis			C-index	AIC
	β	HR (95% CI)	*P* value	β	HR (95% CI)	*P* value		
Age	0.355	1.426 (1.193–1.705)	< 0.001	0.312	1.366 (1.093–1.707)	0.006	0.512	4137.4
Gender	0.128	1.136 (0.922–1.399)	0.230				0.546	4146.5
Tumor location	0.005	1.005 (1.003–1.008)	< 0.001				0.495	4146.4
Macroscopic type	0.587	1.798 (1.629–1.984)	< 0.001	0.174	1.190 (1.018–1.391)	0.029	0.657	4032.1
**Differentiation degree**	0.473	1.606 (1.417–1.819)	< 0.001				0.591	4112.3
Tumor diameter	0.519	1.681 (1.541–1.833)	< 0.001				0.656	4039.4
T stage	0.752	2.121 (1.906–2.359)	< 0.001	0.332	1.394 (1.071–1.815)	0.014	0.686	3979.3
N stage	0.637	1.891 (1.762–2.029)	< 0.001	0.485	1.625 (1.471–1.795)	<0.001	0.728	3919.9
V stage	0.646	1.907 (1.752–2.076)	< 0.001	0.200	1.221 (1.004–1.486)	0.045	0.701	3962.4
Vessel invasion	1.173	3.230 (2.490–4.190)	< 0.001				0.627	4062.3
Neural invasion	1.214	3.366 (2.318–4.887)	< 0.001				0.574	4095.7

HR: Hazard ratio; CI: Confidence interval.

### Comparison of predictive value of V and VNM stage

**Figure 2:**
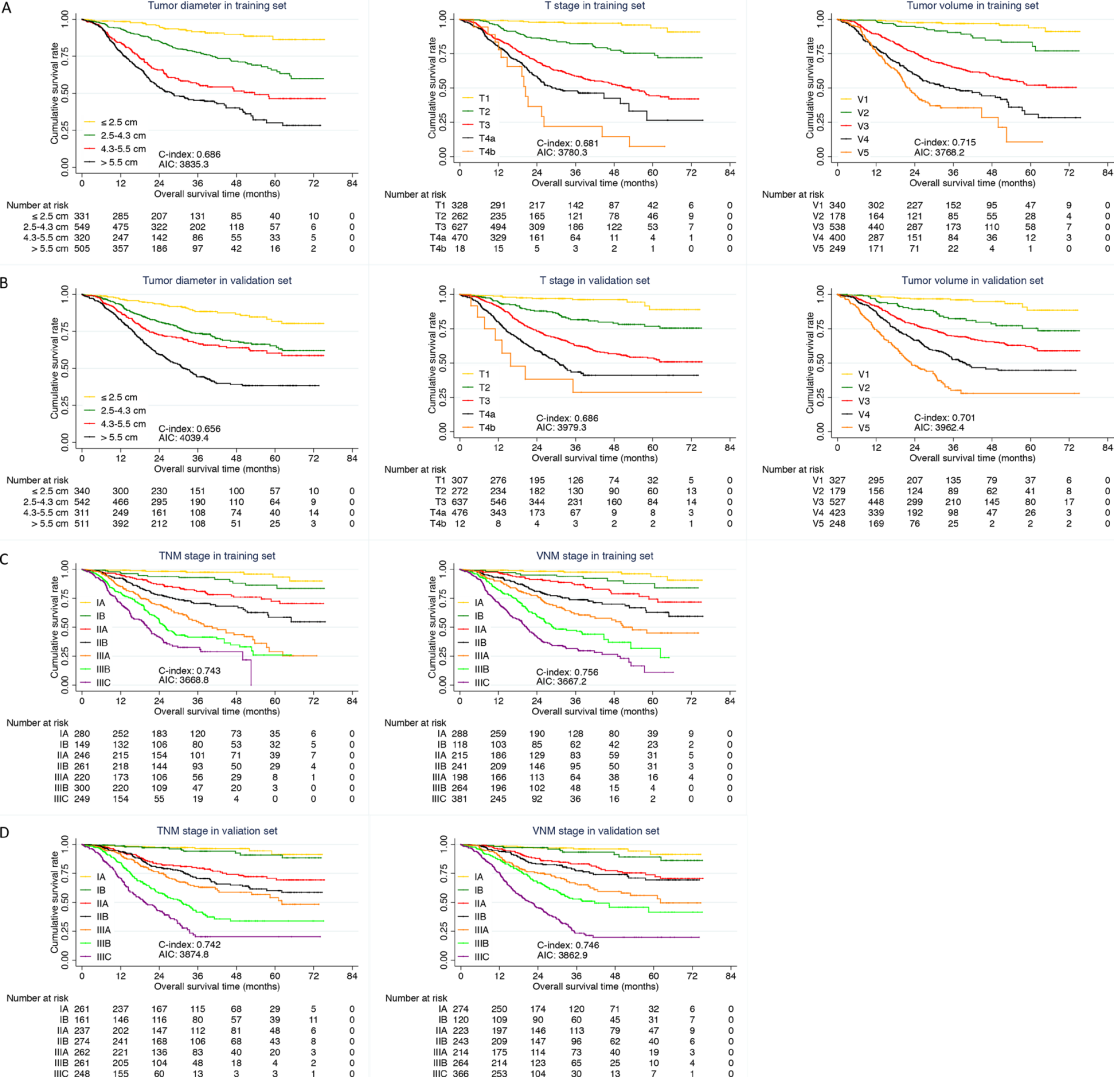
Comparison of predictive value (**A**) Comparison among tumor diameter, T stage and V stage in training set; (**B**) Comparison among tumor diameter, T stage and V stage in validation set; (**C**) Comparison between TNM and VNM stage in training set; (**D**) Comparison between TNM and VNM stage in validation set.

C-index and AIC were calculated in order to assess the predictive accuracy and prognostic discriminatory ability of each factor for prognosis of gastric cancer in training set (Table 2). A larger C-index and smaller AIC value of V stage were found when compared with tumor diameter (C-index: 0.715 vs 0.686; AIC: 3768.2 vs 3835.3, *P <* 0.001) and T stage (C-index: 0.715 vs 0.681; AIC: 3768.2 vs 3780.3, *P <* 0.001) (Figure 2A). VNM stage also revealed significant superiority to TNM stage in predictive accuracy and prognostic discriminatory ability (C-index: 0.756 vs 0.743; AIC: 3667.2 vs 3668.8, *P <* 0.001) (Figure 2C).

In validation set, the predictive accuracy and prognostic discriminatory ability of V stage and VNM stage were still better than that of tumor diameter, T stage (Table 3, Figure 2B) and TNM stage (Figure 2D) respectively.

### Multivariable models and nomograms

Two multivariable prediction models were built in training set. TNM model was based on the selection of age, gender, tumor location, macroscopic type, differentiation degree, T stage, N stage, vessel invasion and neural invasion. VNM model was based on the selection of age, gender, tumor location, macroscopic type, differentiation degree, N stage, V stage, vessel invasion and neural invasion. Finally, results of the two multivariable regression models were showed in Table 4. Consistent with the results of multivariate analysis above, V stage was still selected as an independent prognostic factor in VNM model.

**Table 4:** Multivariable models for predicting overall survival in training set

Characteristics	TNM model			VNM model		
	β	HR (95% CI)	*P* value	β	HR (95% CI)	*P* value
Age	0.307	1.359 (1.080–1.711)	0.009	0.288	1.334 (1.059–1.680)	0.015
Macroscopic type	0.269	1.309 (1.121–1.529)	0.001	0.253	1.288 (1.103–1.503)	0.001
**Differentiation degree**	0.166	1.181 (0.966–1.443)	0.105	0.198	1.219 (1.000–1.487)	0.005
T stage	0.412	1.510 (1.269–1.798)	< 0.001	—	—	—
N stage	0.562	1.754 (1.575–1.954)	< 0.001	0.541	1.719 (1.543–1.913)	< 0.001
V stage	—	—	—	0.331	1.392 (1.223–1.585)	< 0.001
C-index	0.767			0.775		
AIC	3648.7			3635.6		

C-index: Harrell's concordance index; AIC: Akaike Information Criterion;

HR: Hazard ratio; CI: Confidence interval.

**Figure 3:**
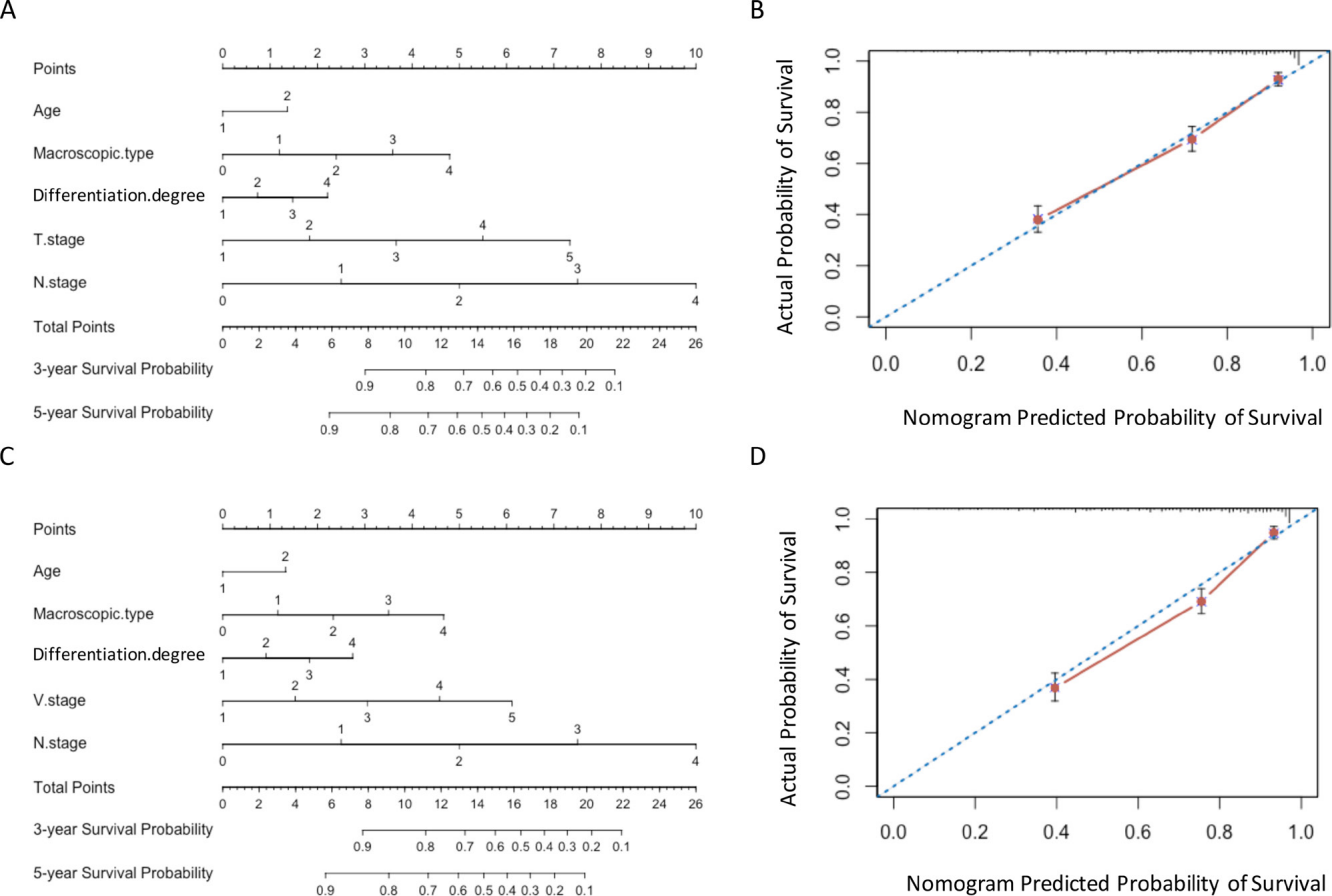
Nomograms in training set (**A**) and (**B**) Nomogram plots and calibration curves of TNM stage; (**C**) and (**D**) Nomogram plots and calibration curves of VNM stage.

Two nomograms were developed for predicting overall survival in training set (Figure 3A and 3C). The VNM model showed significant advantages than TNM model in predictive accuracy and prognostic discriminatory ability (C-index: 0.775 vs 0.767; AIC: 3635.6 vs 3648.7, *P <* 0.001) (Table 4). The calibration curves of the two models both showed good agreement between predicted and actual outcomes (Figure 3B and 3D).

**Figure 4:**
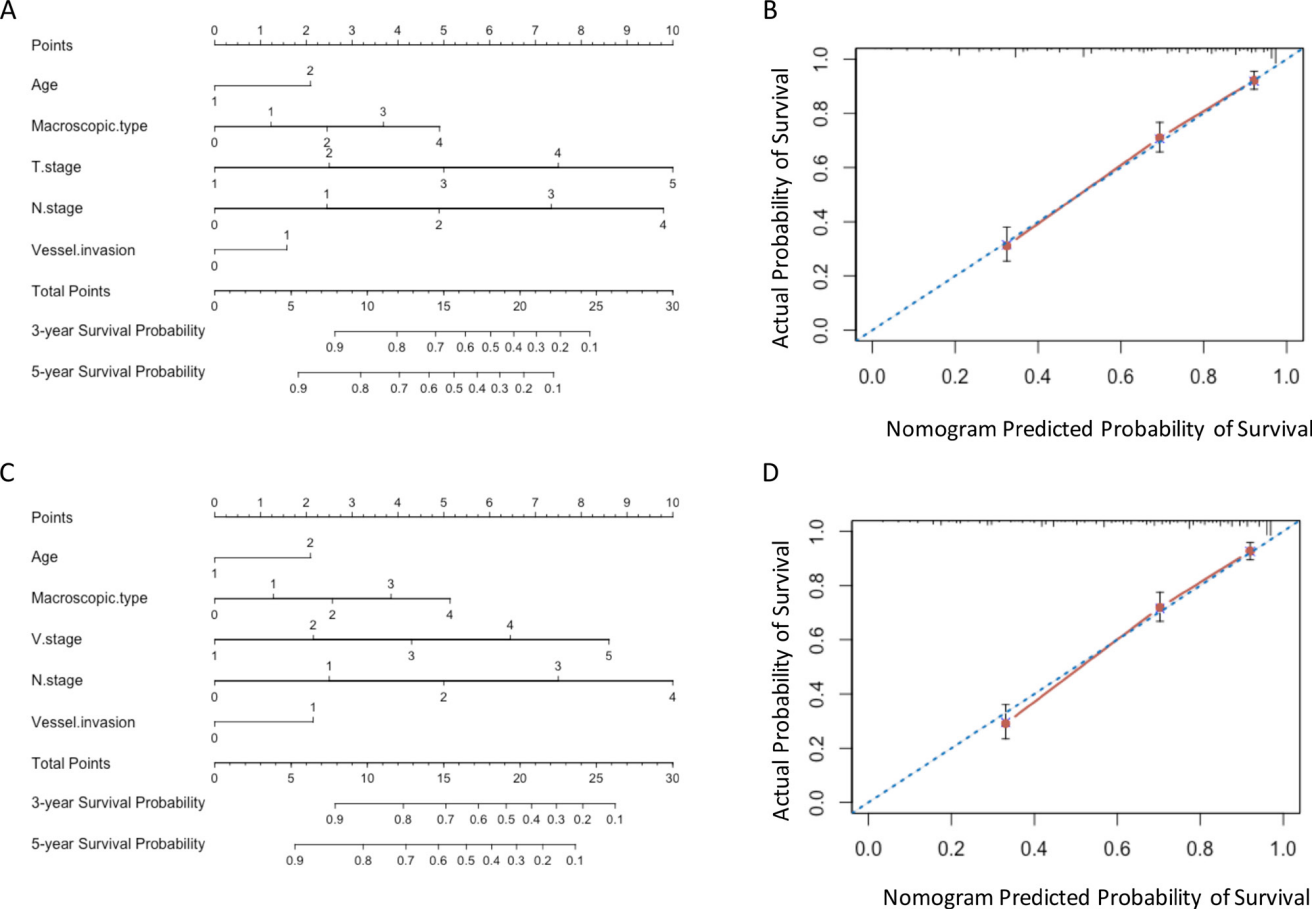
Nomograms in validation set (**A**) and (**B**) Nomogram plots and calibration curves of TNM stage; (**C**) and (**D**) Nomogram plots and calibration curves of VNM stage.

The results in validation set were consistent with those in training set. The predictive accuracy and prognostic discriminatory ability of VNM model were significant better than those of TNM model (Table 5). The predicted survival of the two models showed good agreement with observed survival (Figure 4).

**Table 5:** Multivariable models for predicting overall survival in validation set

Characteristics	TNM model			VNM model		
	β	HR (95% CI)	*P* value	β	HR (95% CI)	*P* value
Age	0.358	1.430 (1.144–1.787)	0.002	0.322	1.380 (1.104–1.726)	0.005
Macroscopic type	0.201	1.223 (1.048–1.427)	0.011	−0.193	1.213 (1.040–1.415)	0.014
Vessel invasion	0.244	1.227 (0.951–1.714)	0.105	0.320	1.378 (1.029–1.844)	0.031
T stage	0.505	1.657 (1.380–1.990)	< 0.001	—	—	—
N stage	0.475	1.607 (1.447–1.785)	< 0.001	0.442	1.556 (1.400–1.730)	< 0.001
V stage	—	—	—	0.379	1.461 (1.276–1.672)	< 0.001
C-index	0.767			0.769		
AIC	3848.6			3848.4		

C-index: Harrell's concordance index; AIC: Akaike Information Criterion;

HR: Hazard ratio; CI: Confidence interval.

### Comparison of formulas

In order to evaluate the superiority of the current volume calculating formula, we further validated the formula reported in the previous study using our center's data (Table 6). The results showed that the V stage, VNM stage and the multivariable model calculated by current formula had a larger C-index and a smaller AIC value than those calculated by the previous formula (all *P <* 0.001).

**Table 6:** Comparison and validation between the two formulas

	Current formula		Previous formula		*P* value
	C-index	AIC	C-index	AIC	
Training group					
V stage	0.715	3768.2	0.693	3845.4	< 0.001
VNM stage	0.756	3667.2	0.732	3753.3	< 0.001
Multivariable model	0.775	3635.6	0.764	3712.6	< 0.001
Validation group					
V stage	0.701	3962.4	0.684	3993.3	< 0.001
VNM stage	0.746	3862.9	0.723	3917.5	< 0.001
Multivariable model	0.769	3848.4	0.756	3908.2	< 0.001

Current formula: V = Tumor diameter × (T stage)^2^/2;

Previous formula [33]: V = pT × (tumor size/2)^2^.

## DISCUSSION

The current study investigated the prognostic value of tumor volume for gastric cancer. The results showed that the predictive value of V stage for gastric cancer was superior to tumor diameter and T stage. VNM staging system could significantly improve the predictive accuracy and prognostic discriminatory ability for gastric cancer.

The actual malignancy of gastric cancer is complex due to the variety of appearances and patterns of invasion [17]. Up to now, T stage and N stage were demonstrated to be the most significant prognostic factors for gastric cancer in several previous studies [18–20]. Tumor diameter, which has been considered as a rough indicator of tumor size for gastric cancer [21, 22], was closely related with histologic type, lymph node metastasis, tumor invasion, vessel invasion, neural invasion and peritoneal metastasis [23–25]. Further investigations demonstrated that tumor diameter was an independent prognostic factor for gastric cancer [26–28]. Saito et al. [28] found that tumor diameter could also be used to predict the recurrence site of gastric cancer. Moreover, Deng et al. [29] demonstrated that tumor diameter represented better prognostic stratification ability compared with T stage, while Zhao et al. [16] reported that the prognostic prediction value was comparable between the two variables. In both studies above, they replaced T stage with tumor diameter in the TNM staging system and found that the new classification was more competent in predicting the prognosis of gastric cancer than the current TNM staging system.

However, tumor diameter or T stage alone could not accurately reflect the actual tumor burden of gastric cancer due to this cancer's complicated morphology and inconsistent pattern of invasion [2, 17, 27, 28]. Thus, a new index which could better reflect the actual size of this tumor is needed.

Tumor volume, which could accurately reflect the tumor burden, may possess significant prognostic value for gastric cancer. Moreover, tumor volume was reported as an independent prognostic factor in several cancers, such as non-small-cell lung carcinoma [30], nasopharyngeal carcinoma [31] and malignant melanoma [32]. However, study assessing the predictive value of tumor volume for gastric cancer is lacking. Up to date, there is only one study reported by Jiang et al [33] that calculated tumor volume via the formula V = pT × (tumor size/2)^2^ demonstrated tumor volume maybe more reliable than T stage in predicting prognosis of gastric cancer in a cohort of 497 patients. Further, they conducted a VNM staging system by replacing the T stage with tumor volume and found that it was more appropriate than the current TNM staging system in predicting prognosis of gastric cancer patients.

In current study, we calculated the tumor volume based on the formula V = Tumor diameter × T stage^2^/2. The mathematic model of tumor volume referred to the formula V = length × width^2^/2 in the tumor bearing mouse model [34]. We used tumor diameter instead of the length and replaced the width with T stage. We first used the C-index and AIC value to evaluate the predictive accuracy and prognostic discriminatory ability for tumor volume, respectively. The predictive value of V stage was higher than tumor diameter and T stage. However, accurate prediction of prognosis is more determined by the staging system than a variable alone [12]. We then conducted the VNM stage on the basis of the two most powerful prognostic predictors—V stage and N stage. The predictive accuracy and prognostic discriminatory ability of VNM stage was better than those of TNM stage.

Further, two nomograms were developed for predicting the overall survival. The VNM model had significant advantages in the predictive accuracy and prognostic discriminatory ability than TNM model. The predicted survival of VNM model showed well agreement with the actual survival.

A good staging system, which could not only be able to predict survival, but also guide the adjuvant therapy, is of great importance for patients with gastric cancer [35]. The predictive superiority of tumor volume demonstrated in current study was consistent with Jiang's findings [33]. To show the improvement we got in this study, we then validated their formula using our data and found that the tumor volume calculated by our formula V = Tumor diameter × T stage2/2 revealed better predictive accuracy and prognostic discriminatory ability.

There are also some limitations in our present study. First, it was a retrospective study of a single center's experiences. Multi-center studies are needed to verify the predictive value of tumor volume. Second, the calculation of tumor volume is not simple and immediate. Thus, a more convenient and accurate index which could reflect the tumor burden is needed.

## MATERIALS AND METHODS

From September 2008 to March 2015, a total of 3409 gastric cancer patients who received radical gastrectomy in our department were retrospectively analyzed. The inclusion criteria were listed as follows: 1) without neoadjuvant chemotherapy; 2) without multiple stomach tumors or distant metastasis; 3) with complete follow-up records. This study was approved by the Ethics Committee of Xijing Hospital, and written informed consent was obtained from all patients before surgery.

All of the patients received radical gastrectomy according to the recommendation of Japanese Gastric Cancer Treatment Guidelines [11]. The patients were followed up till November 2015 by enhanced chest and abdominal CT and gastroscopy every 3 months.

Clinicopathological data including age, gender, tumor location, macroscopic type, tumor diameter, differentiation degree, T stage, N stage, vessel invasion, neural invasion and TNM stage were recorded. Tumor diameter was measured and defined as the maximum diameter of the tumor according to the Japanese classification of gastric carcinoma: 3rd English edition [36]. The TNM stage were defined on the basis of 7th edition of AJCC cancer staging manual [10].

Data were processed using SPSS 22.0 for Windows (SPSS Inc., Chicago, IL, USA). With the X-tile software (Yale University) [37], the 3409 patients were randomly divided into training set and validation set according to sample size ratio of 1:1. The optimal cut-off values of tumor volume were calculated using X-tile software (Supplementary). Discrete variables were analyzed using the Chi-square test or Fisher's exact test. Risk factors for survival were identified by univariate analysis and Cox's proportional hazards regression model was employed for multivariate analysis. Overall survival was analyzed by the Kaplan-Meier method and differences between curves were compared using log-rank test. A backward procedure based on the Akaike information criterion (AIC) was used for multivariable selection. Nomogram and calibration curve were displayed using the package of Regression Modeling Strategies (http://CRAN.R-project.org/package=rms) in R (version3.1.2, http://www.R-project.org/). AIC and concordance index (C-index) values within a cox proportional hazard regression model were calculated in order to compare the prognostic discriminatory ability and predictive accuracy of variables using the package of Harrell Miscellanceous (http://CRAN.R-project.org/package=Hmisc.). A smaller AIC value indicated a better discriminatory ability [38], whereas a larger C-index represented a more predictive accuracy [39]. The likelihood ratio χ2 test was used to compare the different C-indexes between different models. The two-tail *P value* was considered to be statistically significant at the 5% level.

## CONCLUSIONS

Tumor volume was significantly associated with clinicopathological features and prognosis of gastric cancer. The predictive value of tumor volume was higher than tumor diameter and T stage. In comparison with TNM staging system, VNM staging system could improve the predictive accuracy and prognostic discriminatory ability for gastric cancer.

## SUPPLEMENTARY FIGURES



## Abbreviations

V: Tumor volume
TNM: Tumor-nodes-metastasis classification
AJCC: American Joint Committee on Cancer
JGCA: Japanese Gastric Cancer Association
AIC: Akaike information criterion
C-index: Concordance index
