# Supplementary material for: Tumor volume increases the predictive accuracy of prognosis for gastric cancer: A retrospective cohort study of 3409 patients
Source: Oncotarget. 2017 Jan 27;8(12):18968–78. doi: 10.18632/oncotarget.14859 (PMC5386662; doi:10.18632/oncotarget.14859)
Supplement: Supplementary file 1 [file oncotarget-08-18968-s001.pdf]

Tumor volume increases the predictive accuracy of prognosis for gastric cancer: A retrospective cohort study of 3409 patients

Supplementary Materials

The introduction of X-tile

The cut-off values in current study were obtained by X-tile software developed by Yale University [1]. X-tile plots provide a single, global assessment of every possible way of dividing a population into low-level and high-level marker expression. X-tile data are presented in a right triangular grid where each point represents a different cut-point. The intensity of the color of each cutoff point represents the strength of the association. The X-tile software allows the user to move a cursor across the grid and provides an "on-the-fly" histogram of the resulting population subsets along with an associated Kaplan-Meier curve. The X-tile software provides a method of dividing a single cohort into training and validation subsets for *P* value estimation when separate training and validation cohorts are not available. In summary, the X-tile software can provide optimal cut off values, and according to the optimal cut off values, patients are divided into two or three groups which have the most significant difference in prognosis.

REFERENCES

1. Camp RL, Dolled-Filhart M, Rimm DL. X-tile: a new bio-informatics tool for biomarker assessment and outcome-based cut-point optimization. Clin Cancer Res. 2004; 10:7252–9. doi: 10.1158/1078-0432.CCR-04-0713.

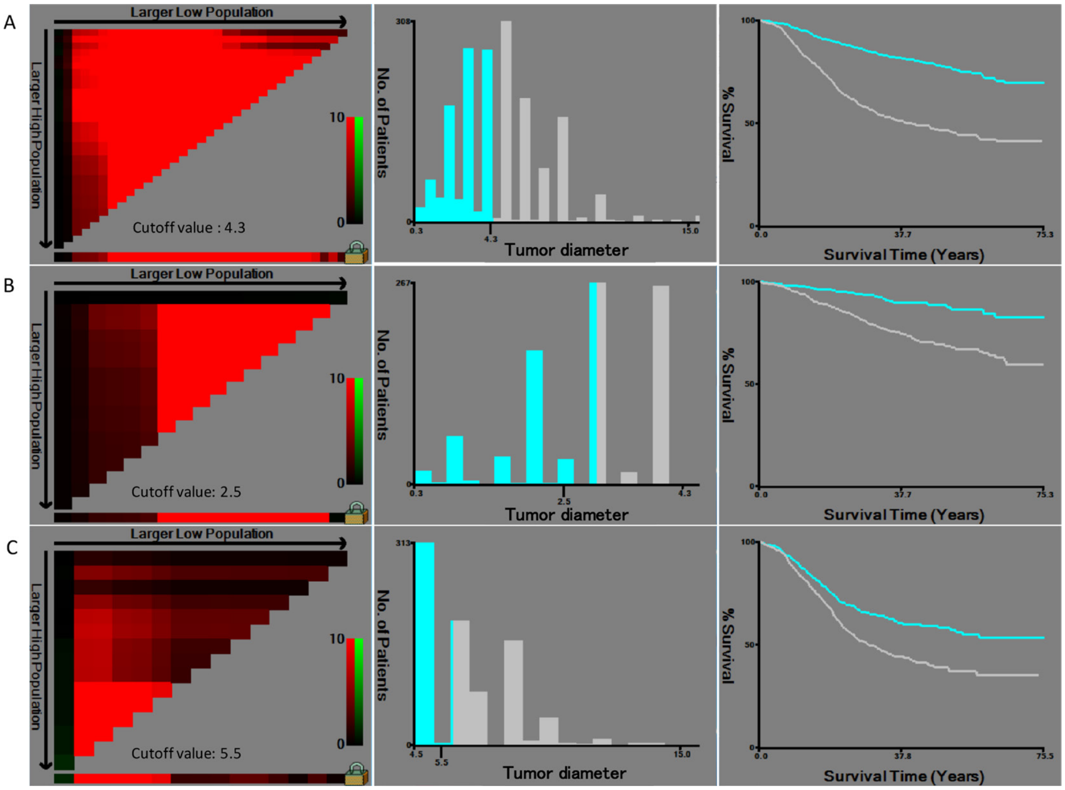

Supplementary Figure 1: Calculation of cutoff points of tumor diameter by X-tile in training set. (**A**) Two subgroups were built according to the optimal cutoff point (4.3, $P < 0.001$); (**B**) Two subgroups were built according to the optimal cutoff point (2.5, $P < 0.001$) for patients with tumor diameter between 0 and 4.3; (**C**) Two subgroups were built according to the optimal cutoff point (5.5, $P < 0.001$) for patients with tumor diameter between 4.3 and 15.0.
